# Biogeography and evolution of the *Carassius auratus*-complex in East Asia

**DOI:** 10.1186/1471-2148-10-7

**Published:** 2010-01-12

**Authors:** Mikumi Takada, Katsunori Tachihara, Takeshi Kon, Gunji Yamamoto, Kei'ichiro Iguchi, Masaki Miya, Mutsumi Nishida

**Affiliations:** 1 Laboratory of Fisheries Biology & Coral Reef Studies, Faculty of Science, University of the Ryukyus, 1 Senbaru, Nishihara, Okinawa 903-0213, Japan; 2 Department of Marine Bioscience, Ocean Research Institute, The University of Tokyo, 1-15-1 Minamidai, Nakano, Tokyo 164-8639, Japan; 3 National Research Institute of Fisheries Science, Fisheries Research Agency, Komaki 1088, Ueda, Nagano 386-0031, Japan; 4 Department of Zoology, Natural History Museum & Institute, Chiba, 955-2 Aoba-cho, Chuo-ku, Chiba 260-8682, Japan

## Abstract

**Background:**

*Carassius auratus *is a primary freshwater fish with bisexual diploid and unisexual gynogenetic triploid lineages. It is distributed widely in Eurasia and is especially common in East Asia. Although several genetic studies have been conducted on *C. auratus*, they have not provided clear phylogenetic and evolutionary descriptions of this fish, probably due to selection bias in sampling sites and the DNA regions analysed. As the first step in clarifying the evolutionary entity of the world's *Carassius *fishes, we attempted to clarify the phylogeny of *C. auratus *populations distributed in East Asia.

**Results:**

We conducted a detailed analysis of a large dataset of mitochondrial gene sequences [*CR*, 323 bp, 672 sequences (528 sequenced + 144 downloaded); *CR *+ *ND4 *+ *ND5 *+ *cyt b*, 4669 bp in total, 53 sequences] obtained from *C. auratus *in East Asia. Our phylogeographic analysis revealed two superlineages, one distributed mainly among the Japanese main islands and the other in various regions in and around the Eurasian continent, including the Ryukyus and Taiwan. The two superlineages include seven lineages with high regional specificity that are composed of endemic populations indigenous to each region. The divergence time of the seven lineages was estimated to be 0.2 million years ago (Mya) by a fossil-based method and 1.0-1.9 Mya by the molecular clock method. The antiquity and endemism of these lineages suggest that they are native to their respective regions, although some seem to have been affected by the artificial introduction of *C. auratus *belonging to other lineages. Triploids of *C. auratus *did not form a monophyletic lineage but were clustered mostly with sympatric diploids.

**Conclusions:**

The results of the present study revealed the existence of two superlineages of *C. auratus *in East Asia that include seven lineages endemic to each of the seven regions examined. The lack of substantial genetic separation between triploids and diploids indicates that triploids are not composed of a single independent lineage. The ancient origins and evolutionary uniqueness of the seven lineages warrant their conservation. An overall phylogenetic framework obtained from the present study will be of use for estimating the phylogenetic relationships of *Carassius *fishes on the Eurasian continent.

## Background

Fish of the genus *Carassius *(Cypriniformes, Cyprinidae), including goldfish, crucian carp, and Japanese crucian carp, primarily inhabit freshwater and are distributed widely in and around the Eurasian continent, including Taiwan and the Japanese islands [1]. Although their classification has not been well established due to their great variability, they can be classified into at least three species: *C. auratus*, *C. carassius*, and *C. cuvieri*. Among them, *C. auratus *is so variable that its varieties are sometimes treated as independent species or subspecies [e.g., *C*. (*a*.) *burgeri*, *C*. (*a*.) *gibelio*, *C*. (*a*.) *grandoculis*, and *C*. (*a*.) *langsdorfii*]. It should be noted that the variability is further enhanced by the existence of goldfish (*C. a. auratus*), which are domesticated ornamental fish that have been produced under artificial selection and are widespread throughout the world. Recently, goldfish were shown to have originated from one group of Chinese *C. auratus *[2]. *Carassius *in and around Japan are very common and variable. Although Japanese *Carassius *have recently been arranged into *C. cuvieri *and five subspecies of *C. auratus *[3], identification of the five subspecies is controversial because of difficulties in distinguishing morphological and molecular characters [4-6].

In addition to the variability described above, the *C. auratus*-complex includes not only bisexual diploid lineages but also unisexual gynogenetic triploid lineages [7-9], and the origin(s) of the triploids is controversial. Some ichthyologists consider each subspecies of *C. auratus *to include both bisexual diploid and gynogenetic triploid lineages [7,10,11], whereas others regard all gynogenetic triploids as belonging to the (sub)species of the Japanese *C*. *auratus langsdorfii *[3,12]. In the latter case, *C*. *a*. *langsdorfii *is considered to be a (sub)species consisting only of triploids with no diploids. To resolve the complicated situations in the natural history and systematics of the fish, one must clarify the biological entities of *C. auratus*, taking into account the existence of triploids.

Genetic and phylogenetic analyses based on DNA information usually are effective in clarifying details about the biological entity of a "species." Phylogenetic analyses of the Japanese *C. auratus*-complex have been conducted using the first third of the control region (CR) of mitochondrial DNA (mtDNA), which is generally the most variable and is thus useful for phylogenetic investigations of closely related organisms [13]. Some of these analyses detected a large degree of genetic divergence between *C. cuvieri *and *C. auratus *[4-6]. Heterogeneity was found within *C. auratus *in Japan, although the five subspecies of *C. auratus *mentioned above could not be discriminated by the analyses based on CR sequences [4,6]. Moreover, it is interesting to note that triploid *C. auratus *is polyphyletic in the Japanese main islands [4-6], as gynogenetic triploid fishes in other groups usually are monophyletic [14]. Despite such efforts in analysing the phylogeny of the complex, well-resolved phylogenetic and evolutionary pictures have not be obtained because of two biases.

The first type of bias involves the selection of sampling sites in island regions in East Asia. These regions are biologically important because they harbour old lineages of various freshwater fishes [e.g., [15-17]. We anticipate that clarifying the biological entity of the *C. auratus*-complex in this region would provide a useful basis for future comprehensive studies of the complex. However, in previous studies of Japanese *C. auratus*, the specimens examined were only collected from the main islands and not from the Ryukyu Archipelago. Obtaining a complete picture of the evolutionary relationships in East Asian *C. auratus *is difficult without specimens from this biogeographically important region, where an old island area intermittently was connected to the Eurasian continent during its long history [18]. Terrestrial and freshwater fauna of the Ryukyu Archipelago are unique in that they include many endemic species and subspecies that have evolved independently on these isolated islands. In total, 1259 (21.4%) of 5887 known terrestrial and freshwater species are endemic to the central and southern Ryukyus [19]. For example, the Ryukyu-ayu, *Plecoglossus altivelis ryukyuensis*, is considered an endemic subspecies in inland waters of the central Ryukyus [20]. In addition, not only primary freshwater fish (e.g., the Japanese rice fish, or medaka, *Oryzias latipes*) but also brackish-water fish (e.g., the Japanese mudskipper, *Periophthalmus modestus*) of the Ryukyu Archipelago are genetically distinct from adjacent conspecific populations [21-23]. Therefore, *C. auratus *of the Ryukyu Archipelago is also expected to have differentiated from other populations. Inspection of *C. auratus *from the Ryukyu Archipelago is crucial in clarifying the phylogenetic picture of *C. auratus *distributed in the island regions of East Asia.

The second bias involves selection of the DNA region analysed. Previous phylogenetic analyses of *C. auratus *mainly used mtDNA, particularly the first third of the CR [e.g., [4-6]. Analyses using maternally inherited mtDNA are highly effective for estimating phylogenetic relationships of organisms, including gynogenetic populations such as *C. auratus*, although they are not as effective in detecting hybridisation conditions [24]. The abundance of published mtDNA sequences also makes mtDNA attractive for phylogenetic analyses of *C. auratus *because these sequences can be used for comparative analyses. However, using the CR is questionable because a "genetic ceiling" of CR sequences has been detected not only at the intrafamily level [25] but also at the intraspecies level [26]. As the possibility of a genetic ceiling of CR sequences in *C. auratus *cannot be excluded, more comprehensive phylogenetic analyses using other mtDNA regions in addition to the CR are required. Although mtDNA may not be necessarily effective in estimating accurate phylogenetic relationship, recent research using AFLP analysis and mtDNA analysis showed that both mitochondrial and nuclear DNAs lead basically the same result in estimating phylogenetic relationships in the *C. auratus*-complex [6].

The present study was conducted to generate phylogenetic and evolutionary pictures of East Asian *C. auratus *populations by eliminating selection bias in sampling sites and mtDNA regions analysed. Specimens of *C. auratus *were collected from the Ryukyu Archipelago in addition to areas in and around the Japanese main islands that have been studied previously. After ploidy determination of the specimens, we sequenced the NADH dehydrogenase subunits 4 (ND4) and 5 (ND5), the cytochrome *b *apoenzyme (cyt *b*) genes, and the CR for phylogenetic analyses using neighbour-joining (NJ), maximum likelihood (ML), and Bayesian inference (BI) methods. The results of these phylogenetic analyses were examined in relation to the geographic distribution of the species. In addition, divergence times between main clades were estimated based on the resulting phylogenetic trees.

## Methods

### Samples

As previously noted, systematics of the genus *Carassius *have not been well established. Because no generally acceptable global classification system for *Carassius *exists, we treated all *C. auratus *specimens (i.e., all *Carassius *specimens excluding *C. carassius *and *C. cuvieri*) as *C. auratus *without further classification.

**Figure 1:**
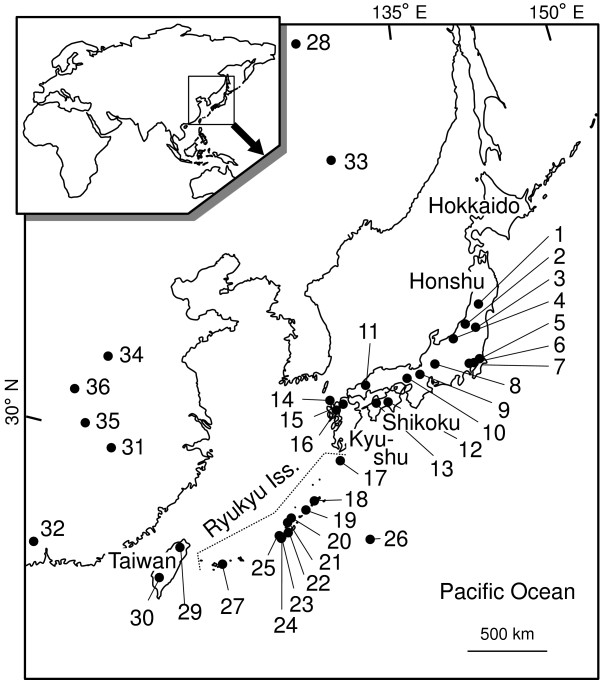
**Map of the sampling sites of *Carassius auratus***. Map showing water systems, islands, and fish markets where *Carassius auratus *specimens were collected. Specimens from waters numbered 1-16, 28, and 31 were collected previously by Yamamoto et al. [6]. Locality numbers correspond to those in Additional file 1 and Figure 6: 1, Lake Sakasazawa; 2, Lake Fukushimagata; 3, Lake Inawashiro; 4, Urano River; 5, Lake Kasumigaura; 6, Magame River; 7, Kamidokan Moat; 8, Nagara River; 9, Lake Biwa; 10, Kako River; 11, Takatsu River; 12, Shimanto River; 13, Shigenobu River; 14, Iki Island; 15, Tatara River; 16, Chikugo River; 17, Tanegashima Island; 18, Amami-oshima Island; 19, Tokunoshima Island; 20, Iheya Island; 21, Izena Island; 22, Okinawa Island; 23, Tokashiki Island; 24, Zamami Island; 25, Kume Island; 26, Minamidaito Island; 27, Ishigaki Island; 28 Amur River; 29, Taipei fish market; 30, Donguan fish market; 31, Yangtze River; 32, Kai Ping; 33, Fangzheng hatchery; 34, Qi hatchery; 35, Jiujiang hatchery; and 36, Wuhan hatchery. The inset shows a map of Eurasia denoting the present sampling area.

We collected 485 *C. auratus *from 11 islands of the Ryukyu Archipelago (Figure 1) using cast-, gill-, and hand-nets between 2003 and 2006. Most specimens were released immediately at the same site after collecting blood or fin samples and sexing as described below. Blood collected from the caudal vein with a small syringe was immediately preserved at -20°C in 99.5% ethanol. Fin clips were also fixed in 99.5% ethanol. In addition, 43 *C. auratus *specimens were bought at two fish markets in Taiwan in 2003, and the collected fin clips were fixed in 99.5% ethanol.

The sex of all specimens collected from the Ryukyu Archipelago was determined. Based on previous observations that all males > 60 mm standard length (SL) from Okinawa Island had pearl organs (small pearly dots on the operculum, fin rays, and scales) and ejected sperm after squeezing the abdominal area throughout the year [27], individuals > 60 mm (SL) with both pearl organs and sperm ejection were considered to be male; all others were considered to be female. All specimens < 60 mm SL were regarded as unknown sex.

In addition to the 528 newly collected specimens (485 from the Ryukyus and 43 from Taiwan), we used 23 fin or muscle tissue samples collected from Honshu, Shikoku, and Kyushu (Japan), Russia, and China [6] deposited at the Ocean Research Institute, the University of Tokyo (ORIUT).

### Ploidy determination

We determined the ploidy of specimens by measuring the relative DNA content of the blood samples by flow cytometry (FCM) using a PAII type flow cytometer (Partec, Münster, Germany). FCM was performed using the manufacturer's protocol with minor modifications. Briefly, 2-5 μl aliquots of blood suspensions were resuspended in 200 μl of extraction buffer (CyStain DNA 2 step: solution A; Partec) and incubated for over 10 min at room temperature. Subsequently, 800 μl of 4', 6-diamidino-2-phenylindole (DAPI) staining solution (CyStain DNA 2 step: solution B; Partec) was added. A sample of blood collected from a single diploid *C. auratus *from the Japanese main islands [6] was used as an internal standard for each FCM profile.

### DNA extraction, PCR amplification and sequencing

Nucleotide sequence data for part of the CR (323 bp) of the mitochondrial genome were obtained from all 528 specimens collected from the Ryukyu Archipelago or purchased at fish markets in Taiwan. These sequences were analysed along with 144 published sequences obtained from a total of 357 *C. auratus *specimens collected from the Japanese main islands [number of individuals (n) = 236], Russia (n = 3), and China (n = 74), along with goldfish (n = 44) [2,6,28]. One specimen was then chosen from each of the different CR haplotypes from a total of 672 sequences (528 sequenced, 144 published), and additional sequence data were obtained from 41 of the 528 newly collected specimens and from 23 of the deposited specimens collected by Yamamoto et al. [6] for the whole ND4 (1381 bp), ND5 (1824 bp), and cyt *b *(1141 bp) genes.

Total genomic DNA was extracted from all specimens using either a DNeasy Tissue Kit (Qiagen, Hilden, Germany) or an Aquapure Genomic DNA Isolation Kit (Bio-Rad, Hercules, CA, USA) in accordance with the manufacturers' protocols. All fragments were amplified by polymerase chain reaction (PCR) with various combinations of 32 primers (see Table 1 for primer sequences) using a model 9700 thermal cycler (Applied Biosystems, Foster City, CA, USA). PCR conditions were as follows: 2 min at 92°C; 30-35 cycles at 94°C for 10-40 sec; 48-55°C for 10-60 sec; 72°C for 60 sec; and a final extension at 75°C for 7 min. For the CR and cyt *b *regions, target segments were amplified directly. For the ND4 and ND5 genes, a segment of about 4000 bp from the ND4L to ND6 genes that spans the ND4 and ND5 genes was amplified. The PCR products were diluted in sterilized TE buffer (1:20) for subsequent use as short PCR templates. All short PCR products were purified using an ExoSAP-IT purification kit (USB Corporation, Cleveland, OH, USA) and then sequenced with dye-labelled terminators (Applied Biosystems) using the same primers as for PCR. All reactions for DNA sequencing were run on ABI 3100 or 3130 sequencers (Applied Biosystems).

**Table 1:** PCR and sequencing primers used in analysis of the *Carassius auratus*-complex mitochondrial genome.

Primer	Sequence (5'→3')
cyt *b*	
L14558-ND6	AGC AAC TAA CCC CAC AAC CA
L14736-Glu	AAC CAC CGT TGT TAT TCA ACT A
L15339-cyt *b*	TTT CTT TCC ACC CAT ACT TTT CA
H14909-cyt *b*	GCG GTT GAA ATG TCT GAG GT
H15913-Thr^a^	CCG GTS TTC GGM TTA CAA GAC CG
H15923-Thr	GGA GCC AGG GGT GAG AGT TA
	
ND4	
L10649_ND4_ca	CTT TTG GCC TTC TCT GCT TG
L10681-ND4-C^b^	GCK TTT TCT GCK TGT GAR GC
L10474-Arg-C^b^	GGT TWG AKT CCG YGG TTC CCT TAT GAC
L11417_ND4_ca	GCA CAT GTA GAA GCC CCT GT
L11427-ND4-C^b^	CCW AAG GCS CAT GTW GAR GC
L12191-His	TTG TGA TTC TAA AAA TAG GGG TTA AA
H11226_ND4_ca	TAA RAG CGG GAG TGA TCC TG
H11618-ND4-C^b^	TGG CTK ACK GAK GAG TAK GC
H11860_ND4_ca	CAG TGG TGG GAG TGC TAG GT
H11875-ND4-tm	AGT TCC CCT ATT AGA TTA GG
H12632-ND5	GAT CAG GTT ACG TAK AGK GC
H12632-ND5-C^b^	TTC TAG GAT KGA TCA GGT GAC GWA KAG KGC
H14710-Glu-C^b^	CTT GTA GTT GAA TWA CAA CGG TGG TTY TTC
	
ND5	
L10474-Arg-C^b^	GGT TWG AKT CCG YGG TTC CCT TAT GAC
L12328-Leu-C^ b^	AAC TCT TGG TGC AAM TCC AAG
L13058-ND5-C^ b^	TCK GCT ATG GAG GGY CCK AC
L13280-ND5M	CAR CTW GGC CTA ATR ATR GT
L13226_ND5_ca	CAC AGC CAC CTG TGC TCT AA
L13559-ND5-C^b^	TCK TAT CTK AAC GCC TGR GC
L13686_ND5_ca	TCC CCA ATT AAC GAA AAY AAT CC
H13393-ND5-C^b^	CCT ATT TTK CGG ATG TCT TGY TC
H13721-ND5-C^b^	ATG CTT CCT CAG GCR AGK CG
H13822_ND5_ca	AGG GTG GYT GGT ATT GTC ATA A
H14225_ND6_ca	GTG ATT TGT GCT TGG GTG CT
H14710-Glu-C^b^	CTT GTA GTT GAA TWA CAA CGG TGG TTY TTC
H14473-ND6-C^b^	GCG GCW TTG GCK GAG CC
	
CR	
L15923^c^	TTA AAG CAT CGG TCT TGT AA
H16500^d^	GCC CTG AAA TAG GAA CCA GA

^a^Mabuchi *et al*. [16]

^b^Miya *et al*. [64]

^c^Iguchi *et al*. [65]

^d^Nishida *et al*. [66]

### Neighbour-joining analysis of the control region

The 528 newly determined CR sequences were analysed together with 100 previously published sequences of the same CR region from 313 *C. auratus *specimens (among which 236 specimens were ploidy-known but the others were unknown) collected from Honshu, Shikoku, Kyushu, Russia, and China [2,6,28] and with the 44 sequences from 44 goldfish specimens collected from Japan and China [2] (see Additional file 1 for accession numbers). In the first step of the analyses, a NJ tree was constructed under the best fit model GTR + *Γ* + *I *[29] in MrModeltest ver. 2.2 [30] using the program PAUP* 4.0b8a [31]. *Cyprinus carpio *{DNA DataBank of Japan (DDBJ)/EMBL Nucleotide Sequence Database (EMBL)/GenBank genetic sequence databank (GenBank) accession number, AP009047; [32]} and *Carassius cuvieri *(AB045144; Murakami, unpublished data) were used as outgroups to root the tree. The reliability of tree nodes was assessed by the bootstrap (BS) method with 1000 replications.

### Maximum likelihood and Bayesian analysis

For the accurate estimation of phylogenetic relationships and divergence time between mtDNA haplotypes, the nucleotide sequences of three protein genes (ND4, ND5, and cyt *b*) were analysed in addition to the CR sequences. Because the rates of nucleotide sequence evolution differ among DNA regions and among codon positions, ML and BI methods that can reflect these differences were used for the analyses. Partitioned ML analysis was conducted with RAxML ver. 7.2.1 [33]. We prepared five datasets (CR, ND4, ND5, cyt *b*, and a concatenated sequence of all four sequences) for analyses and set three partitions (1^st^, 2^nd^, and 3^rd ^codon positions for ND4, ND5, and cyt *b*) or four partitions (1^st^, 2^nd^, and 3^rd ^codon positions and CR for the concatenated sequence) assuming that functional constraints on sequence evolution are more similar within codon positions (or types of molecule) across genes than across codon positions (or types of molecule) within genes. The GTR + *Γ* model [34] (the model recommended by the author of the program) was used and rapid BS analysis was conducted with 1000 replications (-f a option). This option performs BS analysis using GTRCAT, which is the GTR approximation with optimisation of individual per-site substitution rates, and classifies those individual rates into a certain number of rate categories. After implementing the BS analysis, the program uses every 5^th ^BS tree as a starting point to search for the ML tree using the GTR + *Γ* model of sequence evolution and saves the top 10 best-scoring ML trees (fast ML searches). Finally, RAxML calculates better likelihood scores (slow ML searches) for these 10 trees and places BS probabilities on the best-scoring ML tree. *Cyprinus carpio *(AP009047; [32]) and *Carassius cuvieri *(AB045144; Murakami, unpublished data) were used as outgroups to root all ML trees.

BI analyses were performed with MrBayes ver. 3.1.2 [35]. We prepared exactly the same five datasets as those used in ML analyses. Monte Carlo Markov chains under the selected best fit model GTR + *I *([36]; for ND4 and ND5), GTR + *Γ* (for cyt *b*), and GTR + *Γ* + *I *(for CR and concatenated sequences) in MrModeltest ver. 2.2 were run for 2,000,000 generations. Trees and parameters were sampled every 100 generations. We discarded the first 1,000,000 generations (10,000 trees) on each run as "burn-in" after confirming chain stationarity from plots of likelihood against generation. Outgroups were the same as those used in the ML analyses.

### Supermatrix analysis

To conduct a more comprehensive investigation of the phylogenetic relationships among *Carassius *species on a worldwide basis using the best-scoring ML tree topology obtained from the above analysis as a backbone constraint, we downloaded partial mitochondrial sequences of the genus from the databases (DDBJ, EMBL, and GenBank) and concatenated these sequences to the prealigned original dataset in FASTA format for each gene or region. Of the 306 CR, 77 cyt *b*, and 8 ND5 sequences, 122, 23, and 5 were judged to be authentic and reliable with clear locality information with reference to the original paper and preliminary alignment, respectively (Table 2). These sequences were included in the supermatrix analysis and the rest were excluded. Incidentally, these excluded sequences did not change the tree topologies of the resultant supermatrix tree when they were included in the analysis, with an exception of one CR sequence from Kazakhstan, whose phylogenetic position was extremely unstable. The concatenated sequences were subjected to multiple alignment using MAFFT ver. 6 [37] with the default parameters. We imported the aligned sequences into MacClade ver. 4.08 [38] and removed the redundant regions appearing as gaps with slight modifications by eye to correctly reproduce the original alignment. Finally, the aligned sequences from each gene or region were concatenated using MacClade to generate a mitochondrial supermatrix consisting of 71 sequences, including 18 sequences from the databases. The supermatrix was subjected to partitioned ML analysis using RAxML ver. 7.2.1 with the best-scoring ML tree topology from the original dataset used as a backbone constraint (-r option in RAxML). The evolution model used and the BS analysis performed in this analysis were the same as those described in the above ML analyses. Outgroups were also the same as those in the previous ML and BI analyses.

**Table 2:** Source, origin, and accession numbers for mitochondrial DNA sequences of *Carassius *fishes used in the supermatrix analysis.

Reference	Scientific name used in the original paper	Country	Accession number (sequence region)
Gilles *et al*. [67]	*Carassius auratus*	France	AJ388413 (CR)
Kalous *et al*. [49]	*C. carassius*	Czech Republic	DQ399938 (cyt *b*)
		Germany	DQ399917- 19 (cyt *b*)
	*C. gibelio*	Czech Republic	DQ399926- 29, 31, 33- 37, 39, 40 (cyt *b*)
	*C. langsdorfii*	Czech Republic	DQ399930, 32 (cyt *b*)
Li and Gui [28]	*C. auratus gibelio*	China	EF633617-39, 41- 80 (CR)
Haynes *et al*. [68]	*C. auratus*	Australia	EU754018- 20 (CR)
Komiyama *et al*. [2]	*C. auratus auratus*	Japan	AB379916, 19, 23- 54 (CR)
			AB378293, 95, 96, 98, 99 (ND5)
		China	AB379955- 59 (CR)
	*C. gibelio*	China	AB377293- 99, AB379922 (CR)
Sakai *et al*. [69]	*C. gibelio gibelio*	Kazakhstan	AB274414- 16 (CR)
Tsipas *et al*. [70]	*C. gibelio*	Greece	EU186831- 35 (CR)
			EU186830, DQ868876- 79 (cyt *b*)

### Divergence time estimation

To obtain a rough estimate of the divergence times of major cladogenic events in *C. auratus*, we used two different procedures. The first method uses the molecular clock. Divergence times were calculated per clade according to the evolutionary rate of 1.52% (pairwise distance) per million year (My) estimated for cyt *b *sequences of cyprinid fish by Zardoya and Doardio [39]. The second method uses the fossil record. The concatenated ML tree obtained was transformed into an ultrametric tree using a non-parametric rate smoothing (NPRS) algorithm [40] in TreeEdit ver. 1.0 [41]. The branches of the NPRS tree were scaled using a divergence time of approximately 0.5 million years ago (Mya) for the *C. cuvieri *lineage based on the oldest fossil record from the Sakawa clay stratum [42].

## Results

### Ploidy and sex of specimens from the Ryukyu Archipelago

The sex of 338 of the 485 specimens collected from the Ryukyus was determined successfully (267 females, 71 males). Ploidy of 436 specimens was also determined (273 diploids, 150 triploids, and 13 tetraploids). Both sex and ploidy of a total of 311 specimens were determined (124 diploid females, 66 diploid males, and 121 triploid females; Table 2). No triploid males were found, and all 13 tetraploids were too small to determine their sex. Diploids were observed from 10 of 11 islands studied, triploids from six islands, and tetraploids from two islands (Table 3).

**Table 3:** List of studied islands in the Ryukyus, collection dates, and numbers of diploid, triploid, tetraploid, and ploidy not determined *Carassius auratus *specimens.

Name of islands	Collection dates	Number of individuals (female/male/not determined)				
		
		Total	Diploid	Triploid	Tetraploid	Not determined
Tanegashima Is.	Sep. 2005	50	8/6/23	0/0/13	-	-
Amami-oshima Is.	July 2004, Dec. 2005	21	8/2/0	11/0/0	-	-
Tokunoshima Is.	Nov. 2005	4	2/2/0	-	-	-
Iheya Is.	Sep. 2003	32	16/3/13	-	-	-
Izena Is.	Oct. 2003	34	26/6/2	-	-	-
Okinawa Is.	Apr. 2003 - Sep. 2004	271	54/38/32	91/0/6	0/0/1	22/5/22
Kume Is.	Oct. 2004	27	8/6/0	13/0/0	-	-
Zamami Is.	Oct. 2005	27	0/0/8	0/0/7	0/0/12	-
Tokashiki Is.	Aug. 2003	1	0/1/0	-	-	-
Minamidaito Is.	Oct. 2003	9	-	6/0/3	-	-
Ishigaki Is.	Aug. 2003	9	2/2/5	-	-	-

Total		485	124/66/83	121/0/29	0/0/13	22/5/22

### CR haplotype phylogenies

DNA segments of 323 bp of the CR were newly sequenced and aligned for the 528 *C. auratus *specimens (485 from the Ryukyus, 43 from Taiwan). A total of 66 nucleotide positions (20.6% in 323 bp) varied, and these variations defined 35 haplotypes. Of these haplotypes, four were identical to previously reported haplotypes [2,6], and the other 31 were newly detected (see Additional file 1 for accession numbers). In addition to the 35 haplotypes, 29 sequences previously obtained in our laboratory [6], six sequences of *C. auratus *collected from China [2,28], and one goldfish sequence [2] were added, providing a total of 71 CR haplotypes for phylogenetic analyses.

**Figure 2:**
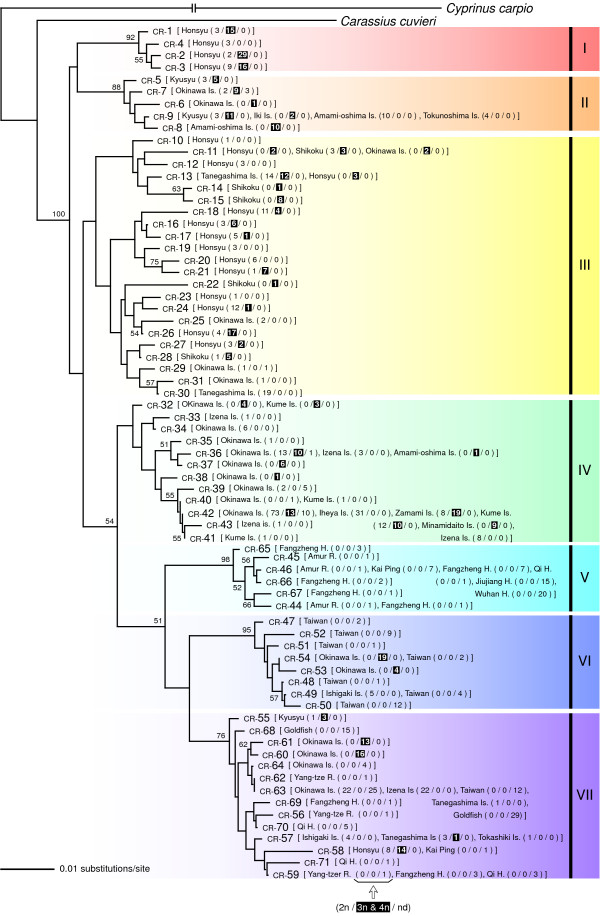
**Neighbour-joining (NJ) tree based on the control region (CR) sequences**. NJ tree of haplotypes detected in *Carassius auratus *based on the CR sequences of mtDNA. Numbers above the internal branches are bootstrap values (< 50% support). Haplotype numbers are followed by the names of islands or rivers where specimens were collected; the number of diploids, triploids + tetraploids (shaded), and individuals for which ploidy was not determined are shown in parentheses. Roman numerals on the right side of the tree denote clade code numbers.

The NJ tree based on the GTR + *Γ* + *I *model demonstrated that the 71 CR haplotypes were divided into seven major clades (I -VII) based average GTR + *Γ* + *I *distances of 0.034-0.098 (Figure 2; Additional file 2). Both diploids and triploids were observed in all major clades, with the exception of clade V, which was composed only of specimens of unknown ploidy. Furthermore, of the 71 haplotypes, 21 were shared by both diploids and triploids in five major clades (see Figure 2 for details), although 14 and 11 haplotypes consisted of only diploids and triploids, respectively.

**Figure 3:**
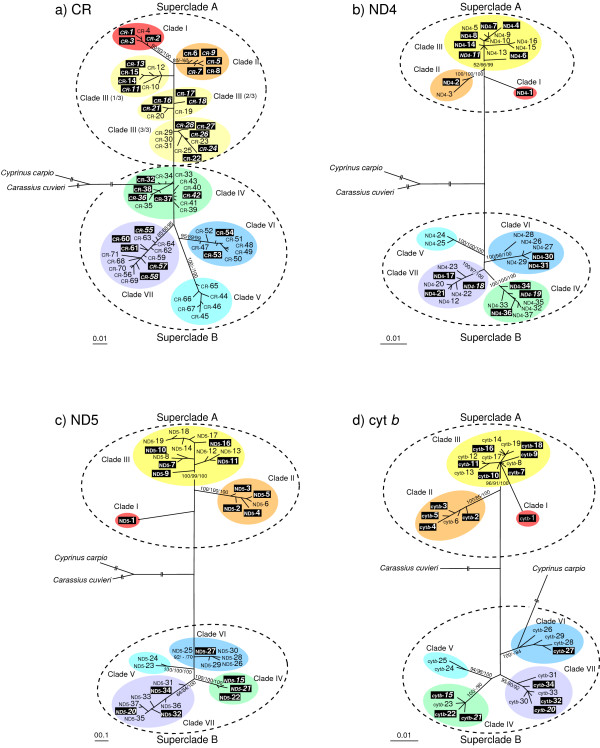
**Maximum likelihood (ML) trees based on the control region (CR), ND4, ND5 and cyt *b *sequences**. The best-scoring maximum likelihood tree from partitioned ML analyses based on the CR (a), ND4 (b), ND5 (c) and cyt *b *(d) sequences of mtDNA. Numbers above the internal branches indicate neighbour-joining and ML bootstrap (BS) values and Bayesian (BI) posterior probabilities. The BS and BI values are given only for the nodes of major clades. Haplotypes observed in triploids are shaded. Haplotypes shared between diploids and triploids are shown in italic and shaded. Clade numbers I - VII correspond to those given in the NJ tree (Figure 2).

ML and BI trees based on the CR data showed an essentially similar haplotype grouping as that of the NJ tree (Figure 3a, only the ML tree is shown). However, the ML and BI tree topologies differed somewhat from that of the NJ tree; clade III detected in the NJ tree was not detected in the ML and BI trees.

### ND4, ND5 and cyt *b *haplotype phylogenies

DNA samples were available for further sequencing for 64 out of 71 CR haplotypes. Complete ND4, ND5, and cyt *b *genes (1381 bp, 1824 bp, and 1141 bp, respectively) were successfully sequenced for 53 of 64 specimens chosen from each of the 64 CR haplotypes. One hundred and sixty three (ND4, 11.8%), 224 (ND5, 12.3%), and 146 (cyt *b*, 12.8%) variable sites without insertions or deletions were found, which defined 37, 37, and 34 haplotypes, respectively, from each of the 53 sequences.

NJ, ML, and BI trees based on each of the three protein-coding regions showed the same seven major clades as those detected in the CR-based NJ tree (Figure 3b-d, only ML trees are shown). These seven clades were grouped into two superclades: A (clades I- III) and B (clades IV-VII). As expected from the results of CR-based NJ analysis, sharing of a haplotype by diploids and triploids was observed in all three protein-coding regions (three haplotypes for ND4, three for ND5, and four for cyt *b*).

**Figure 4:**
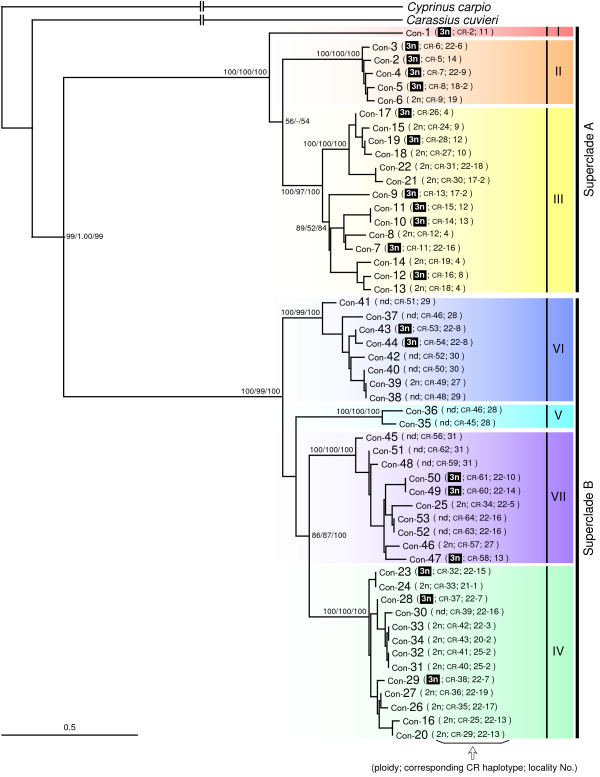
**Maximum likelihood (ML) tree based on the concatenated sequences of the control region (CR), ND4, ND5 and cyt *b***. The best-scoring ML trees from partitioned ML analysis based on a 4669 bp concatenated sequence of the four mtDNA regions. Numbers above the internal branches indicate neighbour-joining and ML bootstrap (BS) values and Bayesian (BI) posterior probabilities. The BS and BI values are given only for the nodes of the major clades. Haplotype numbers of concatenated sequences are followed by ploidy [2n, diploid; 3n, triploid (shaded); nd, ploidy not determined], corresponding CR haplotype numbers, and locality numbers. See Figure 6 for locality names. Roman numerals on the right side of the tree denote clade code numbers.

Because mtDNA comprises a single circular molecule, it is preferable to use the 4669 bp concatenated sequence of all four regions (CR, ND4, ND5, and cyt *b*) to estimate the complete mtDNA phylogeny. NJ and best-scoring ML and BI trees based on the concatenated sequence corroborated the existence of the seven major clades and the two superclades revealed in the analyses described above, with higher BS values (100%) and BI posterior probabilities (100%; Figure 4, only the ML tree is shown). These seven major clades were separated by average GTR + *Γ* + *I *distances of 0.019-0.103 (Additional file 3).

### Supermatrix analysis

**Figure 5:**
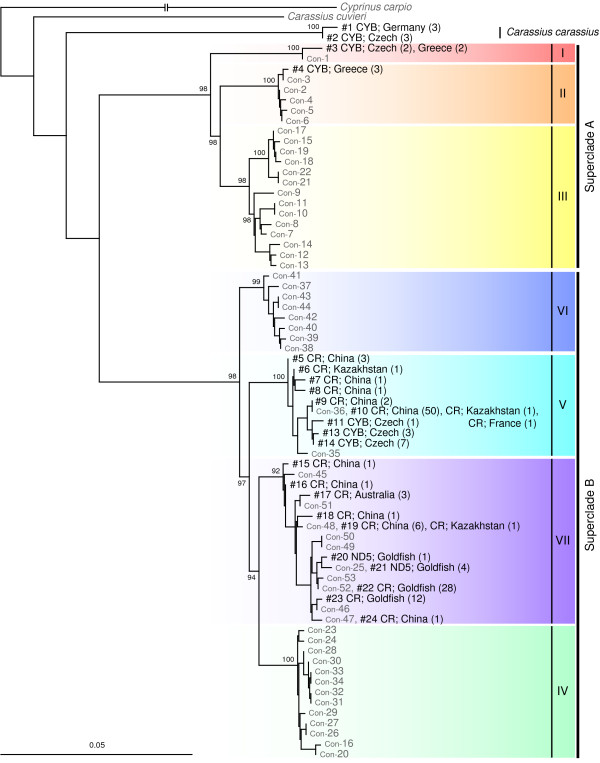
**Supermatrix tree of *Carassius auratus *with additional sequences**. Maximum likelihood (ML) supermatrix tree generated using the best-scoring concatenated ML tree (Figure 4) as a backbone constraint. Accession numbers of the sequences are as follows: #1, DQ399917-19; #2, DQ399938; #3, DQ399930, DQ399932, DQ868879, EU186830; #4, DQ868876-78; #5, EF633624, EF633626, EF633633; #6, AB27415; #7, EF633617; #8, EF633622; #9, EF633625, EF633634; #10, EF633621, EF633627-32, EF633642, EF633646-80, AB377293-99, AB273316, AJ388413; #11, DQ399926; #12, DQ399927, DQ399933-34; #13, DQ399929, DQ399931, DQ399935-47, DQ399940; #14, EF633620; #15, EF633638; #16, EU754018-20; #17, EF633637; #18, EF633618-19, EF633623, EF633635-36, AB274414; #19, AB378296; #20, AB378293, AB378295, AB378298-99; #21, AB379916, AB379919, AB379923-29, AB379931-33, AB379935-41, AB379945-46, AB379948-50, AB379952, AB379957-58; #22, AB379930, AB379934, AB379942-44, AB379947, AB379951, AB379953-54, AB379956, AB379959; #23, AB379922. Numbers above the internal branches are bootstrap values, which are given only for the nodes of major clades. Haplotype numbers of concatenated sequences correspond to those in Figure 4. Haplotype numbers of newly added sequences are followed by the names of the regions of mitochondrial DNA sequences, locality, and number of sequences. Roman numerals on the right side of the tree denote clade code numbers.

The results of supermatrix analysis performed using the best-scoring ML tree topology as a backbone constraint are shown in Figure 5. All newly downloaded sequences of *C. auratus *from around the world were nested in either of the seven major clades detected in our analyses; these sequences did not form a new major clade. *Carassius carassius *was placed as a sister group of the *C. auratus*-complex.

### Distribution pattern analysis of CR haplotypes

To clarify the relationships between the phylogenetic and geographical structures of *C. auratus *populations, we analysed the distribution pattern of haplotypes within each of the seven major clades. For this analysis, we focused on CR haplotypes because these contained the greatest number of specimens (528 + 357 = 885) and examined their geographical distribution in seven regional areas: Honshu, Shikoku, Kyushu, the Ryukyus, Russia, Taiwan, and China, defined based on relative isolation. As a result, in many major clades the distribution of the haplotypes was restricted to specific area(s) (Figure 5). In particular, haplotypes of clades I, IV, and V were found only in Honshu, the Ryukyus, and the continental region including Russia and China, respectively. Similarly, haplotypes in clades II, III, and VI were distributed mainly in Kyushu, Honshu + Shikoku, and Taiwan, respectively, although some haplotypes of the three clades were found in the Ryukyus (i.e., outside of their main distribution areas). Although haplotypes in clade VII were distributed over a wide geographical range, more than half (7/12) the haplotypes observed in China (CR-56, 58, 59, 62, and 69 - 71) belonged to this clade.

**Figure 6:**
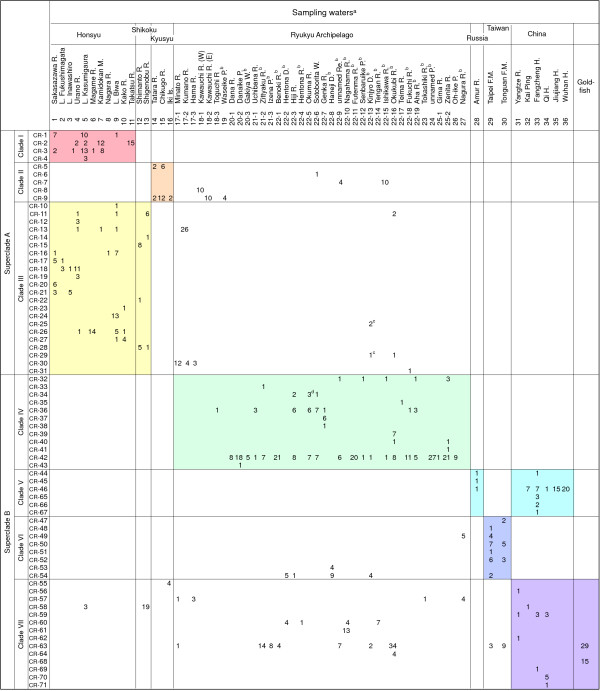
**Geographical distributions of the control region (CR) haplotypes**. Geographical distributions of individuals with each CR haplotype (see Figure 2). Shading indicates the putative natural distribution area of each clade. Locality numbers given to sampling sites correspond to those in Additional file 1 and Figure 1. (a) R., river; L., lake; M., moat; Is., island; P., pond; W., waterway; D., dam; Re., artificial reservoir; F. M., fish market. (b) Water systems with artificial reservoir(s). (c) Analysis of the three mtDNA genes suggested that these three specimens appeared to have haplotypes belonging to clade IV. (d) Analysis of the three mtDNA genes suggested that one of the three specimens appeared to have a haplotype belonging to clade VII.

Note that most haplotypes found outside of their main distribution areas were observed in distinctive water systems (i.e., systems with artificial reservoir(s)). For example, three haplotypes (CR-49, 53, and 54) in clade VI that are typical of Taiwan (see Discussion) were found in 5 of the 38 water systems sampled in the Ryukyus, and 4 of these systems have artificial reservoirs (Figure 6). In addition, seven haplotypes (CR-55, 57, 58, 60, 61, 63, and 64) in clade VII that seemed to be indigenous to China (see Discussion) were observed in Honshu (from 1 of 11 water systems), Shikoku (1/2), Kyushu (1/3), the Ryukyus (14/38), and Taiwan (2/2). Many of these water systems inhabited by clade VII haplotypes were also distinctive (1 in Honshu, 12 in the Ryukyus, and 2 in fish markets in Taiwan); the one in Honshu was Lake Kasumigaura, which is well known for the surfeit of artificially introduced alien fish. In addition, the 12 water systems of the Ryukyus have artificial reservoirs (Figure 6).

### Divergence time estimation

Based on a cyt *b *calibration using the molecular clock value of 1.52%/My, divergence time between superclades A and B was estimated to be about 4.0 Mya and between the seven major clades about 1.0-1.9 Mya (Table 4). When we applied the divergence time of approximately 0.5 Mya for the *C. cuvieri *lineage to the NPRS ultrametric tree based on the best ML tree derived from the concatenated sequence data set, the divergence time between superclades A and B was estimated to be about 0.4 Mya (Table 4). The divergence time among the seven major clades within superclades A and B was around 0.2 Mya, ranging from only 0.17 to 0.21 Mya.

**Table 4:** Estimated date of separation of the major clades of the *Carassius auratus*-complex.

	Based on cyt *b *sequences (1141 bp)		Based on NPRS tree of the concatenated sequences of all four regions (4669 bp)
		
Cladogenetic event	Average *P *distances	**Branching date (Mya) estimated from the molecular clock (1.52%/My) obtained by Zardoya and Doardio **[39]	Branching date (Mya) estimated from the fossil record of *Carassius cuvieri*
Between superclades A and B	0.061	4.01	0.39
			
Within superclade A			
Between clade I and clades II+III	0.029	1.91	0.20
Between clades II and III	0.027	1.78	0.17
			
Within superclade B			
Between clade VI and clades IV+V+VII	0.020	1.32	0.21
Between clade V and clades IV+VII	0.017	1.12	0.19
Between clade IV and VII	0.015	0.99	0.17

## Discussion

### MtDNA trees of East Asian *C. auratus*

In this study, we examined mtDNA to study the phylogeny of the island region-centred East Asian *C. auratus*-complex. We analysed three protein-coding genes in addition to the CR, the latter of which has been used in previous phylogenetic analyses of these fish [e.g., [4-6]. NJ, ML, and BI analyses based on the concatenated sequence (4669 bp) all produced essentially the same tree topology. Most nodes were supported by high NJ and ML BS values and BI posterior probabilities. Considering the robust tree topology obtained from all analyses, we considered these mtDNA trees to adequately reflect the phylogeny of East Asian *C. auratus*.

CR tree topologies differed from that of the concatenated tree in that the two superclades (A and B) were not well separated; this discrepancy might have been caused by a genetic ceiling, probably due to saturation, as in the Chaetodontidae and *Plecoglossus altivelis *[25,26]. In addition, whereas the first third of the CR was more variable than the three protein-coding genes (20.6% vs. 11.8%, 12.3%, and 12.8%, respectively), the total number of variable sites was considerably smaller in the former than in the latter genes (66 vs. 163, 224, and 146, respectively). It seems that the first third of the CR was effective in detecting each lineage in our NJ analysis but was less effective in resolving branching order. Longer sequences of protein-coding gene(s) must be used for an accurate analysis of *C. auratus *phylogeny, although the first third of the CR is effective and useful for identifying mtDNA haplotypes.

### Evolutionary relationships of East Asian *C. auratus*

The resultant mtDNA tree obtained from the 4669 bp concatenated sequence has important implications for the evolutionary relationships of East Asian *C. auratus *(Figure 4). One important implication is the existence of two old superclades with high regional specificity. Superclade A consists of clades I-III, which are distributed mainly in the Japanese main islands, and superclade B consists of clades IV-VII, which are distributed in various regions in and around the Eurasian continent. Divergence time estimation between superclades A and B using the NPRS tree calibrated with the fossil record of *C. cuvieri *(0.4 Mya) and cyt *b *calibration using the molecular clock value of 1.52% per My (4.0 Mya) suggests that the two superclades evolved independently for a considerable period of time in the Japanese main islands and the Eurasian continent, respectively (Table 4). Difference in divergence times estimated by both procedures may be partially explained by the fact that the fossil-based divergence time is the minimum estimation. The Japanese main islands are known to have been inhabited by old lineages of various freshwater fishes [e.g.,[15-17]. Superclade A of *C. auratus *represents one such example of old lineages that is harbored in this island region.

Another important implication is the existence of seven clades: three clades (I -III) within superclade A and four clades (IV-VII) within superclade B. The seven clades diverged greatly from one another, and their origins were estimated to be old (0.2 Mya by the fossil-based method and 1.0-1.9 Mya by molecular clock method; Table 4). These clades showed rather high regional specificities (Figure 6). Such high regional specificities and ancient origins suggest that each of the seven clades represents the natural population of each region. Considering the possibilities of artificial introduction of *C. auratus *noted below, we concluded that *C. auratus *with mtDNA of each of the clade I-VII haplotypes represents the natural population of Honshu (I), Kyushu (II), Honshu + Shikoku (III), the Ryukyus (IV), Russia + China (V), Taiwan (VI), and China (VII), respectively.

### Derivation of regional populations

The results of the present study indicate the existence of seven regional endemic populations distributed in Honshu, Kyushu, Honshu + Shikoku, the Ryukyus, Russia + China, Taiwan, and China. Some individuals belonging to clades II, III, VI, and VII, however, were collected from outside the regions specific to these lineages. These individuals could be regarded as offspring of artificially introduced *C. auratus *for the following reasons: (i) the occurrence of such individuals was mostly sporadic; (ii) despite the ancient origin of the lineages and their geographic isolation, some individuals shared the same haplotype (CR-11, 49, and 54) with fish from inside their original region; and (iii) in the Ryukyus, 128 of 134 (95%) individuals with haplotypes specific to Taiwan and China were collected from water systems with artificial reservoir(s) where freshwater fishes tend to be released. Assuming that the individuals collected from outside of the region specific to the lineages were derived from artificial introduction, five introduction routes can be identified: from the Eurasian continent to the Japanese main islands (route 1); from the Eurasian continent to the Ryukyus (route 2); from the Eurasian continent to Taiwan (route 3); from the Japanese main islands to the Ryukyus (route 4); and from Taiwan to the Ryukyus (route 5). Four of the five routes (routes 1, 3, 4, and 5) are supported by the presence of records for past introductions of freshwater fish [43-45].

### A biogeographic perspective of Eurasian *C. auratus*

In the present study, the phylogenetic picture of *C. auratus *distributed in the Eurasian continent was obtained by supertmatrix analysis using the best-scoring ML tree topology as a backbone constraint. This analysis is very useful for estimating phylogenetic relationships among specimens by adding any portion of the DNA sequences used in the analysis generating the backbone constraint with a reliable phylogenetic tree topology. Based on the results of the analysis, our mtDNA tree was judged to be a good reflection of the overall framework of the phylogenetic entity for the *Carassius *fishes around the world because all of the newly downloaded sequences collected from various geographical regions were included in either of the seven major clades detected in our phylogenetic analyses and no other new major lineage was detected (Figure 5, see also Methods). Our sampling in East Asian regions may sufficiently cover most lineages of *C. auratus*.

The present supermatrix analysis provided important insights into the origin of European *C. auratus*. The major haplotypes observed in Europe were those of clade V. Although a haplotype observed in France might be derived from artificial introduction in that this haplotype was shared by specimens collected from Russia, China, and Kazakhstan (haplotype #10 of the supermatrix tree; Figure 5), those observed in the Czech Republic are considered to be native to Europe because these cyt *b *sequences form a monophyletic lineage (Figure 5), with an estimated divergence time of 0.2 Mya (0.004 in *P *distance) based on cyt *b *calibration. Clade V is major in Eastern Eurasia as well, indicating that this clade must have an extensive distribution on the Eurasian continent.

**Figure 7:**
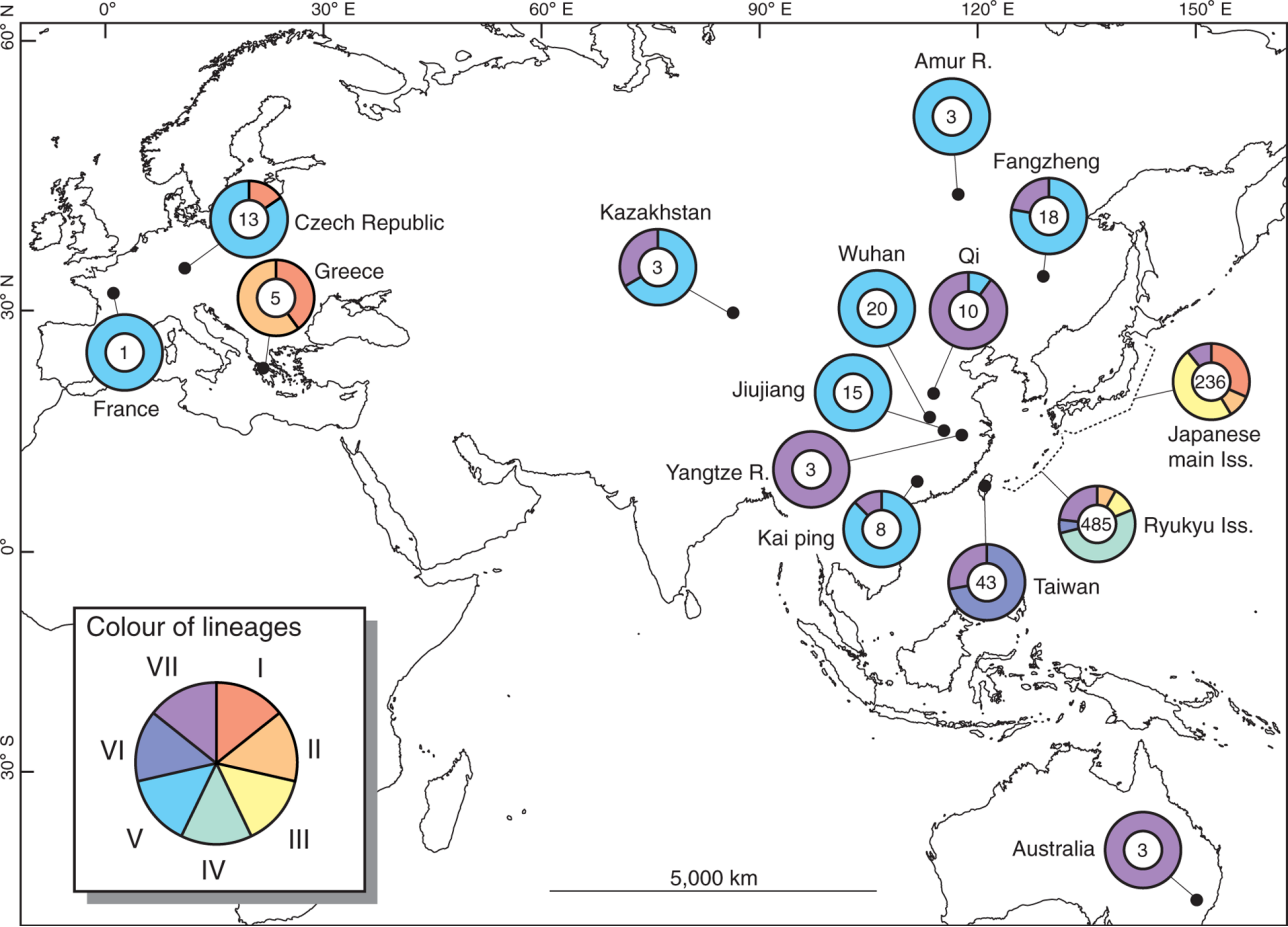
**Global geographical distribution of seven major lineages**. Geographical distributions of haplotypes of the seven major lineages found in the present phylogenetic analyses and supermatrix analysis. Number in each pie graph denotes the number of sequences examined in the supermatrix analysis.

All haplotypes other than those of clade V in European *C. auratus *were nested in Japanese native lineages (clades I and II). These clade I and II haplotypes were found only from the Czech Republic and Greece on the Eurasian continent, whereas clade I and II haplotypes were predominant in the Japanese main islands (Figure 7). This distributional disjunction leads to the hypothesis that European fish with these haplotypes originated via artificial introduction(s) from Japan. Artificial introduction of *Carassius *fishes has been a problem in the European region [46-50], and release of ornamental goldfish into natural waters is considered to be one of the primary causes of dispersion of non-native *C. auratus*. However, European *C. auratus *included in the present study may not have been derived from the release of ornamental goldfish because the goldfish is clearly a member of the Chinese *C. auratus *(clade VII) and has diverged greatly from specimens of clade I, II, and V haplotypes (Figures 2 and 6, and also see Komiyama et al. [2]). Much more sequence data for *C. auratus *collected throughout Eurasia is needed for more detailed phylogenetic and evolutionary pictures of the *C. auratus*-complex.

### Phylogeny of triploid *C. auratus*

Our study also has significant implications for the phylogenetic entity of triploid *C. auratus*. All triploids examined in the present study were female, which is consistent with the idea that triploids reproduce gynogenetically [51-53]. Gynogenetic polyploid lineages in fish generally are considered to have arisen by hybridisation between two related bisexual species, and these polyploid lineages usually can be traced to a single or a few maternal founders [14]. In fact, most known gynogenetic polyploid fish are confirmed to have originated via hybridisation [54]. For triploid *C. auratus*, some authors have considered the possibility of a hybrid origin, although paternal species were unknown [55,56]. In the present study, however, most triploid *C. auratus *were sympatric with diploids; they shared the same haplotype with sympatric diploids more frequently than with allopatric triploids (Figure 2). In addition, triploids were not monophyletic, and no substantial genetic separation between triploids and diploids was observed. These results strongly imply multiple origins of triploid *C. auratus*, as suggested previously [4,56]. Our results can be explained if triploids sometimes arise or have arisen recently from sympatric diploids and vice versa.

Such interconversion of diploid and triploid forms may be very rare in fish, and only one example is known for cyprinids: the *Leuciscus alburnoides *complex. In this complex, ploidy forms differ from each other in genome constitution, and ploidy interconversion occurs through hybridogenesis [57,58]. Whether similar mechanisms also exist in *C. auratus *is not clear. Our preliminary analysis of the nuclear genome of *C. auratus *suggested that no characteristic exists that distinguishes triploids from diploids [6], whereas other studies have suggested the existence of markers mostly specific to triploids [56,59,60]. Comprehensive investigation of nuclear and mitochondrial genomes should be conducted to clarify the origin of triploids and the process and mechanisms of possible ploidy interconversion in *C. auratus*.

### Conservation of endemic lineages of Japanese Archipelago

As discussed above, at least seven lineages of *C. auratus *are indigenous to various regions of East Asia. The populations of the four endemic lineages (Honshu, Kyushu, Honshu + Shikoku, and the Ryukyus) of the Japanese Archipelago can be regarded as evolutionarily significant units (ESU; [61]) due to their high phylogenetic independence and evolutionary distinctiveness. Therefore, conservation of these lineages is necessary. Moreover, note that distribution areas and population sizes of the Ryukyuan *C. auratus *lineage in particular are decreasing rapidly, and some island populations are becoming endangered [62]. These reductions appear to be caused mainly by habitat degradation due to simplification of stream morphology through development. To conserve *C. auratus *populations in the Ryukyus, the main stream habitats must be preserved.

Artificial introduction of non-native *C. auratus *may cause another problem: genetic disturbance of native populations. *Carassius *fishes have been artificially introduced both intentionally and unintentionally in several ways [46-50]. In Japan, many *C. auratus *have been imported alive to the Ryukyus from Taiwan for food (O. Kuniyoshi, pers. comm.). In addition, as suggested by Ohara [63], the possibility of accidental introduction of *C. auratus *exists during seed release of aquacultured fish, such as the Japanese crucian carp, *C. cuvieri*. Once *C. auratus *is introduced into natural waters, either intentionally or unintentionally, genetic disturbance of endemic populations may occur immediately by hybridisation between indigenous and introduced individuals. Careless transplantation of *C. auratus *should be prevented to conserve the genetic uniqueness of the endemic populations.

## Conclusions

Phylogenetic analyses based on large data sets of mitochondrial gene sequences (4669 bp) obtained from the East Asian *Carassius auratus*-complex revealed the existence of two superlineages, one distributed mainly in the Japanese main islands and the other in various regions in and around the Eurasian continent. The two superlineages include seven old (0.2 Mya) lineages endemic to different regions, although some have been affected by artificial introduction of *C. auratus *from other regions. The present analyses provided an overall phylogenetic framework for *C. auratus *that can be used in to estimate the phylogenetic relationships of *C. auratus *on the Eurasian continent. Triploids of *C. auratus *did not form a monophyletic lineage but instead clustered mostly with sympatric diploids, indicating that they are not composed of a single independent lineage. The ancient origins and evolutionary uniqueness of these lineages warrant their conservation.

## Authors' contributions

MT, KT, and MN designed the study; MT, GY, and KI collected the specimens; and MT and GY conducted the molecular work. MT and MM analysed the data. MT, TK, and MN drafted the original manuscript, and KT, KI, and MM contributed to the improvement of the manuscript. All authors read and approved the final manuscript.

## Supplementary Material

Additional file 1 **Accession numbers for all sequences used in this study**. Selected specimens used for sequencing on ND4, ND5, and cyt *b *genes and accession numbers for all sequences used in this study. File format:.pdf Click here for file

Additional file 2 **Average genetic distances within and between seven major clades in the control region (CR)**. Average pairwise *P *distance (above the diagonal) and GTR + *Γ *+ *I *distances (below the diagonal) in the mitochondrial CR of the *Carassius auratus*-complex within and between the seven major clades. File format:.pdf Click here for file

Additional file 3 **Average genetic distances within and between the seven major clades in concatenated sequences**. Average pairwise *P *distance (above the diagonal) and GTR + *Γ *+ *I *distances (below the diagonal) in the concatenated sequences of mitochondrial control region, ND4, ND5, and cyt *b *regions of the *Carassius auratus*-complex within and between the seven major clades. File format:.pdf Click here for file
