# Supplementary material for: Biogeography and evolution of the Carassius auratus-complex in East Asia
Source: BMC Evol Biol. 2010 Jan 12;10:7. doi: 10.1186/1471-2148-10-7 (PMC2820001; doi:10.1186/1471-2148-10-7)
Supplement: Additional file 1 — Accession numbers for all sequences used in this study. Selected specimens used for sequencing on ND4, ND5, and cyt b genes and accession numbers for all sequences used in this study. File format:.pdf [file 1471-2148-10-7-S1.PDF]

| Specimens | | | Mitochondrial DNA regions[a] | | | | | | | | |
|---|---|---|---|---|---|---|---|---|---|---|---|
| | | | CR (323 bp) | | ND4 (1381 bp) | | ND5 (1824 bp) | | Cyt *b* (1141 bp) | | Concatenated (4669 bp) |
| Catalog[b] No. | Locality[f] | Ploidy[g] | Haplo-type No. | Accession No. | Haplo-type No. | Accession No.[r] | Haplo-type No. | Accession No.[r] | Haplo-type No. | Accession No.[r] | Haplotype No. |
| - | | - | CR-1 | AB079921[h] | - | | - | | - | | - |
| ORIUT-T01115[c] | 11 | 3n | -2 | AB079930[h] | ND4-1 | AB368603 | ND5-1 | AB368640 | cyt *b*-1 | AB368677 | Con-1 |
| - | | - | -3 | AB079922[h] | - | | - | | - | | - |
| - | | - | -4 | AB079929[h] | - | | - | | - | | - |
| ORIUT-T01101[c] | 14 | 3n | -5 | AB079965[h] | -2 | AB368604 | -2 | AB368641 | -2 | AB368678 | -2 |
| URM-P44008 | 22-6 | 3n | -6 | AB368568 | -2 | (AB368604) | -3 | AB368642 | -3 | AB368679 | -3 |
| ORIUT-T01625[d] | 22-9 | 3n | -7 | AB368569 | -2 | (AB368604) | -4 | AB368643 | -4 | AB368680 | -4 |
| ORIUT-T01626[d] | 18-2 | 3n | -8 | AB368570 | -2 | (AB368604) | -5 | AB368644 | -5 | AB368681 | -5 |
| ORIUT-T01632[d] | 19 | 2n | -9 | AB368571 | -3 | AB368605 | -6 | AB368645 | -6 | AB368682 | -6 |
| - | | - | -10 | AB079915[h] | - | | - | | - | | - |
| ORIUT-T01622[d] | 22-16 | 3n | -11 | AB368572 | -4 | AB368606 | -7 | AB368646 | -7 | AB368683 | -7 |
| ORIUT-T01046[c] | 4 | 2n | -12 | AB079953[h] | -5 | AB368607 | -8 | AB368647 | -8 | AB368684 | -8 |
| ORIUT-T01631[d] | 17-2 | 3n | -13 | AB368573 | -6 | AB368608 | -9 | AB368648 | -9 | AB368685 | -9 |
| ORIUT-T01603[c] | 13 | 3n | -14 | AB079955[h] | -7 | AB368609 | -10 | AB368649 | -10 | AB368686 | -10 |
| ORIUT-T01600[e] | 12 | 3n | -15 | AB079958[h] | -7 | (AB368609) | -10 | (AB368649) | -10 | (AB368686) | -11 |
| ORIUT-T01598[e] | 8 | 3n | -16 | AB079901[h] | -8 | AB368610 | -11 | AB368650 | -11 | AB368687 | -12 |
| - | | - | -17 | AB079979[h] | - | | - | | - | | - |
| ORIUT-T01048[c] | 4 | 2n | -18 | AB079951[h] | -9 | AB368611 | -12 | AB368651 | -12 | AB368688 | -13 |
| ORIUT-T01040[c] | 4 | 2n | -19 | AB079952[h] | -10 | AB368612 | -13 | AB368652 | -13 | AB368689 | -14 |
| - | | - | -20 | AB079978[h] | - | | - | | - | | - |
| - | | - | -21 | AB079980[h] | - | | - | | - | | - |
| - | | - | -22 | AB079962[h] | - | | - | | - | | - |

| | | | | | | | | | | | |
|---|---|---|---|---|---|---|---|---|---|---|---|
| - | | - | -23 | AB079971[h] | - | | - | | - | | - |
| ORIUT-T01599[c] | 9 | 2n | -24 | AB079908[h] | -11 | AB368613 | -14 | AB368653 | -14 | AB368690 | -15 |
| ORIUT-T01612[d] | 22-13 | 2n | -25 | AB368574 | -12 | AB368614 | -15 | AB368654 | -15 | AB368691 | -16 |
| ORIUT-T01070[c] | 4 | 3n | -26 | AB079903[h] | -11 | (AB368613) | -16 | AB368655 | -16 | AB368692 | -17 |
| ORIUT-T01094[c] | 10 | 2n | -27 | AB079906[h] | -13 | AB368615 | -17 | AB368656 | -17 | AB368693 | -18 |
| ORIUT-T01601[e] | 12 | 3n | -28 | AB079956[h] | -14 | AB368616 | -16 | (AB368655) | -18 | AB368694 | -19 |
| ORIUT-T01611[d] | 22-13 | 2n | -29 | AB368575 | -12 | (AB368614) | -15 | (AB368654) | -15 | (AB368691) | -20 |
| ORIUT-T01630[d] | 17-2 | 2n | -30 | AB368576 | -15 | AB368617 | -18 | AB368657 | -19 | AB368695 | -21 |
| ORIUT-T01624[d] | 22-18 | 2n | -31 | AB368577 | -16 | AB368618 | -19 | AB368658 | -19 | (AB368695) | -22 |
| ORIUT-T01629[d] | 22-15 | 3n | -32 | AB368578 | -17 | AB368619 | -15 | (AB368654) | -15 | (AB368691) | -23 |
| ORIUT-T01618[d] | 21-1 | 2n | -33 | AB368579 | -18 | AB368620 | -15 | (AB368654) | -15 | (AB368691) | -24 |
| ORIUT-T01609[c] | 22-5 | 2n | -34 | AB368580 | -19 | AB368621 | -20 | AB368659 | -20 | AB368696 | -25 |
| ORIUT-T01617[d] | 22-17 | 2n | -35 | AB368581 | -20 | AB368622 | -15 | (AB368654) | -15 | (AB368691) | -26 |
| URM-P43987 | 22-19 | 2n | -36 | AB368582 | -12 | (AB368614) | -15 | (AB368654) | -15 | (AB368691) | -27 |
| ORIUT-T01615[d] | 22-7 | 3n | -37 | AB368583 | -18 | (AB368620) | -21 | AB368660 | -21 | AB368697 | -28 |
| ORIUT-T01613[d] | 22-7 | 3n | -38 | AB368584 | -21 | AB368623 | -22 | AB368661 | -22 | AB368698 | -29 |
| ORIUT-T01614[d] | 22-16 | nd | -39 | AB368585 | -22 | AB368624 | -21 | (AB368660) | -23 | AB368699 | -30 |
| ORIUT-T01621[d] | 25-2 | 2n | -40 | AB368586 | -23 | AB368625 | -21 | (AB368660) | -21 | (AB368697) | -31 |
| ORIUT-T01628[d] | 25-2 | 2n | -41 | AB368587 | -23 | (AB368625) | -21 | (AB368660) | -21 | (AB368697) | -32 |
| ORIUT-T01627[d] | 22-3 | 2n | -42 | AB368588 | -18 | (AB368620) | -21 | (AB368660) | -21 | (AB368697) | -33 |
| ORIUT-T01610[d] | 20-2 | 2n | -43 | AB368589 | -18 | (AB368620) | -21 | (AB368660) | -21 | (AB368697) | -34 |
| - | | - | -44 | AB080010[h,I] | - | | - | | - | | - |
| ORIUT-T01608[c] | 28 | nd | -45 | AB080011[h] | -24 | AB368626 | -23 | AB368662 | -24 | AB368700 | -35 |
| ORIUT-T01607[c] | 28 | nd | -46 | AB080009[h,j] | -25 | AB368627 | -24 | AB368663 | -25 | AB368701 | -36 |
| ORIUT-T01635[c] | 30 | nd | -47 | AB368590 | -26 | AB368628 | -25 | AB368664 | -26 | AB368702 | -37 |
| ORIUT-T01634[c] | 29 | nd | -48 | AB368591 | -27 | AB368629 | -26 | AB368665 | -27 | AB368703 | -38 |
| URM-P43991 | 27 | 2n | -49 | AB368592 | -27 | (AB368629) | -26 | (AB368665) | -28 | AB368704 | -39 |
| ORIUT-T01636[c] | 30 | nd | -50 | AB368593 | -27 | (AB368629) | -26 | (AB368665) | -29 | AB368705 | -40 |
| ORIUT-T01633[c] | 29 | nd | -51 | AB368594 | -28 | AB368630 | -27 | AB368666 | -27 | (AB368703) | -41 |
| ORIUT-T01637[c] | 30 | nd | -52 | AB368595 | -29 | AB368631 | -28 | AB368667 | -27 | (AB368703) | -42 |
| ORIUT-T01623[d] | 22-8 | 3n | -53 | AB368596 | -30 | AB368632 | -29 | AB368668 | -27 | (AB368703) | -43 |

| | | | | | | | | | | | |
|---|---|---|---|---|---|---|---|---|---|---|---|
| URM-P44010 | 22-8 | 3n | -54 | AB368597 | -31 | AB368633 | -30 | AB368669 | -27 | (AB368703) | -44 |
| - | | - | -55 | AB079963[h] | - | | - | | - | | - |
| ORIUT-T01605[e] | 31 | nd | -56 | AB080013[h] | -32 | AB368634 | -31 | AB368670 | -30 | AB368706 | -45 |
| URM-P43993 | 27 | 2n | -57 | AB368598 | -33 | AB368635 | -32 | AB368671 | -31 | AB368707 | -46 |
| ORIUT-T01602[c] | 13 | 3n | -58 | AB079923[h,k] | -34 | AB368636 | -33 | AB368672 | -32 | AB368708 | -47 |
| ORIUT-T01606[e] | 31 | nd | -59 | AB080014[h,l] | -35 | AB368637 | -34 | AB368673 | -33 | AB368709 | -48 |
| URM-P44000 | 22-14 | 3n | -60 | AB368599 | -36 | AB368638 | -35 | AB368674 | -34 | AB368710 | -49 |
| ORIUT-T01616[d] | 22-10 | 3n | -61 | AB368600 | -36 | (AB368638) | -35 | (AB368674) | -34 | (AB368710) | -50 |
| ORIUT-T01604[e] | 31 | nd | -62 | AB080012[h] | -37 | AB368639 | -36 | AB368675 | -33 | (AB368709) | -51 |
| ORIUT-T01620[d] | 22-16 | nd | -63 | AB368601[i,m] | -19 | (AB368621) | -20 | (AB368659) | -20 | (AB368696) | -52 |
| ORIUT-T01619[d] | 22-16 | nd | -64 | AB368602 | -19 | (AB368621) | -37 | AB368676 | -20 | (AB368696) | -53 |
| - | | - | -65 | EF633624[h,n] | - | - | - | - | - | - | - |
| - | | - | -66 | EF633625[h,o] | - | - | - | - | - | - | - |
| - | | - | -67 | EF633622[h] | - | - | - | - | - | - | - |
| - | | - | -68 | AB379917[h,p] | - | - | - | - | - | - | - |
| - | | - | -69 | EF633620[h] | - | - | - | - | - | - | - |
| - | | - | -70 | EF633638[h,q] | - | - | - | - | - | - | - |
| - | | - | -71 | EF633637[h] | - | - | - | - | - | - | - |

[a] CR, control region; ND 4 and 5, NADH dehydrogenase 4 and 5; cyt *b*, cytochrome *b*

[b] ORIUT, Ocean Research Institute, University of Tokyo; URM, Department of Marine Science, University of Ryukyus

[c] catalog numbers for muscle samples

[d] catalog numbers for blood samples

[e] catalog numbers for fin samples

[f] see Fig. 5 for locality names

[g] 2n, diploid; 3n, triploid; nd, ploidy not determined

[h] accession numbers of sequences downloaded (otherwise, newly sequenced in this study)

[i] only the accession number of one representative sequence is shown because 323 bp sequences of other one specimen (EF633617) was the same with this sequence

[j] only the accession number of one representative sequence is shown because 323 bp sequences of other 50 specimens (EF633621, 27- 32, 42, 46- 80) were the same with this sequence

[k] only the accession number of one representative sequence is shown because 323 bp sequences of other one specimen (AB379922) was the same with this sequence

[l] only the accession number of one representative sequence is shown because 323 bp sequences of other six specimens (EF633618, 19, 23, 35, 36, 39) were the same with

this sequence

[m] only the accession number of one representative sequence is shown because 323 bp sequences of other 29 specimens (AB379915, 16, 19, 23-29, 31-33, 35-41, 45, 46, 48-50, 52, 55, 57, 58) were the same with this sequence

[n] only the accession number of one representative sequence is shown because 323 bp sequences of other 2 specimens (EF633626, 33) were the same with this sequence

[o] only the accession number of one representative sequence is shown because 323 bp sequences of other one specimen (EF633634) was the same with this sequence

[p] only the accession number of one representative sequence is shown because 323 bp sequences of other 14 specimens (AB379918, 20, 21, 30, 34, 42-44, 47, 51, 53, 54, 56, 59) were the same with this sequence

[q] only the accession number of one representative sequence is shown because 323 bp sequences of other four specimens (EF633641, 43- 45) were the same with this sequence

[r] accession numbers in parentheses are those already described in the same column
