# Supplementary material for: Biogeography and evolution of the Carassius auratus-complex in East Asia
Source: BMC Evol Biol. 2010 Jan 12;10:7. doi: 10.1186/1471-2148-10-7 (PMC2820001; doi:10.1186/1471-2148-10-7)
Supplement: Additional file 2 — Average genetic distances within and between seven major clades in the control region (CR). Average pairwise P distance (above the diagonal) and GTR + Γ + I distances (below the diagonal) in the mitochondrial CR of the Carassius auratus-complex within and between the seven major clades. File format:.pdf [file 1471-2148-10-7-S2.PDF]

Clade number	I	II	III	IV	V	VI	VII
I	0.005<br>0.005	0.027	0.345	0.042	0.056	0.056	0.058
II	0.034	0.006<br>0.007	0.032	0.046	0.053	0.063	0.06
III	0.046	0.042	0.021<br>0.026	0.034	0.048	0.054	0.054
IV	0.058	0.066	0.043	0.01<br>0.01	0.037	0.035	0.028
V	0.088	0.08	0.07	0.049	0.006<br>0.007	0.044	0.037
VI	0.083	0.098	0.077	0.044	0.057	0.007<br>0.007	0.035
VII	0.092	0.097	0.081	0.035	0.05	0.046	0.008<br>0.008
