# Supplementary material for: Biogeography and evolution of the Carassius auratus-complex in East Asia
Source: BMC Evol Biol. 2010 Jan 12;10:7. doi: 10.1186/1471-2148-10-7 (PMC2820001; doi:10.1186/1471-2148-10-7)
Supplement: Additional file 3 — Average genetic distances within and between the seven major clades in concatenated sequences. Average pairwise P distance (above the diagonal) and GTR + Γ + I distances (below the diagonal) in the concatenated sequences of mitochondrial control region, ND4, ND5, and cyt b regions of the Carassius auratus-complex within and between the seven major clades. File format:.pdf [file 1471-2148-10-7-S3.PDF]

Clade number	I	II	III	IV	V	VI	VII
I	-<br>-	0.028	0.026	0.059	0.059	0.059	0.058
II	0.034	0.002<br>0.002	0.02	0.055	0.053	0.054	0.053
III	0.032	0.024	0.008<br>0.009	0.054	0.054	0.055	0.054
IV	0.1	0.09	0.087	0.002<br>0.002	0.02	0.019	0.017
V	0.103	0.088	0.089	0.024	0.004<br>0.004	0.022	0.02
VI	0.1	0.087	0.089	0.022	0.026	0.004<br>0.004	0.019
VII	0.1	0.087	0.087	0.019	0.024	0.022	0.004<br>0.004
